# Application of Stem Cells and Advanced Materials in Nerve Tissue Regeneration

**DOI:** 10.1155/2018/4243102

**Published:** 2018-09-16

**Authors:** Huaqiong Li, Adam Qingsong Ye, Ming Su

**Affiliations:** ^1^School of Biomedical Engineering, School of Ophthalmology & Optometry and Eye Hospital, Wenzhou Medical University, Wenzhou 325035, China; ^2^School of Dentistry, The University of Queensland, Herston 4006, Australia; ^3^Department of Chemical Engineering, Northeastern University, Boston, MA 02115, USA

Tissue engineering is a field that combines engineering and life science principles to bring about a betterment to humanity through cellular or tissue function reconstitution. With this in mind, two main components of materials and cell sources are usually included in optimizing tissue engineering constructs. Engineered neural graft integrates advanced biomaterials (including various bioactive factors), and suitable stem cells can be orchestrated to facilitate nerve tissue repair and regeneration. This special issue addresses the role of stem cells and biomaterials (including growth factors) in neural tissue regeneration. The themes include scaffold design, signaling pathway study, different stem cell assessments, and various nerve tissue regeneration applications, such as brain injury repair, cerebral ischemia-reperfusion injury repair, and spinal cord injury repair. From 10 submissions, 6 papers are published in this special issue. Each paper was reviewed by at least two reviewers and revised according to reviewers' comments. The papers cover the following neural repair and regeneration strategies: seeding of appropriate stem cells only and integration of biomaterials (including synthetic biopolymers, hydrogels, and growth factors) and stem cells.

During the last decade, tremendous progress has been achieved in stem cell biology and its application in treating various disorders of neural systems. In particular, the multipotency and easy access to large quantity of specific target cell offer unique opportunities for neural tissue regeneration. In this special issue, prospects and emerging challenges of using different stem cell-based therapies on common neurological diseases were discussed. Dental pulp stem cells (DPSCs) is one kind of mesenchymal stem cells (MSCs) and possess MSC-like characteristics such as the ability for self-renewal and multilineage differentiation. In addition, DPSCs which originated from the cranial neural crest express neuron-related markers even before being induced to neuronal differentiation. Taken together, these unique properties make DPSCs an excellent candidate for stem cell-related therapies in nerve diseases. In L. Luo et al.'s review article, the authors summarized the neuronal differentiation potential, neurotrophic, angiogenic, and immunomodulatory properties of DPSCs in the pathological and injured nervous system. This paper reports that DPSCs have the biological properties of MSCs and possess a considerable capacity to differentiate into neuron-like cells and secrete neuron-related trophic factors due to their cranial neural crest origin. DPSCs also can express neuronal markers without preinduced differentiation. Meanwhile, DPSCs can both directly and indirectly stimulate formation of new blood vessels and enhance blood flow to injury sites because of its vascularization and immunomodulatory properties. Thus, both nondifferentiated and differentiated DPSCs are emerging as new cell sources for the treatment of nervous system deficits associated with SCI, stroke, AD, PD, and long gaps of peripheral nerve injury.

In Y. Liu et al.'s paper, menstrual blood-derived endometrial stem cells (MenSCs) were successfully isolated and applied to possible nerve tissue regeneration. Firstly, MenSCs were characterized to show their multipotency and immunophenotype. An upregulation of N-cadherin (N-cad) expression at mRNA and protein levels was detected after transdifferentiation into glial-like cells, when MenSCs were cultured and induced *in vitro*. Moreover, *in vivo* studies also clearly showed that the knockdown of N-cad by in utero electroporation perturbed the migration and maturation of mouse neural precursor cells (NPCs). Taken together, the paracrine effect of MenSCs on neuroprotection and their potential of transdifferentiation into glial-like cells were confirmed. This study implies that transgenic MenSC-based therapy could be a potential tool for peripheral nerve injury repair.

In H. Yang et al.'s paper, the authors explored the effects of multiple tail vein injections of NSC-CM (conditioned medium derived from neural stem cells) on cerebral ischemia-reperfusion (I/R) rat model. They found that NSC-CM significantly ameliorated neurological defects and reduced cerebral infarct volume, accompanied by preserved mitochondria ultrastructure. Significant cell apoptosis of NSC-CM in ischemic hemisphere via improving the expression of Bcl-2 (B-cell lymphoma-2) was found too. This work suggested that NSC-CM might be an alternative and effective therapeutic intervention for ischemic stroke.

Spinal cord injury (SCI) is an intractable challenge with limited treatments in the world. Induced pluripotent stem cell-derived neural stem/progenitor cell (iPSC-NS/PCs) transplantation has been shown as a promising treatment for SCI; however, these applications are still vulnerable which may lead to tumorigenicity. Tumorigenicity of iPSC-NS/PCs is comprised of two aspects: teratoma and true tumor formation. Recent studies revealed that manifold factors had participated into the process of tumorigenesis including the use of tumor-inducing reprogramming factors and residual undifferentiated cells. Of note, the change of epigenetics played a pivotal role in iPSC-NS/PCs tumorogenesis. In J. Deng et al.'s review article, the authors summarized the recent studies regarding iPSC-NS/PC tumorigenicity in the treatment of SCI. Two different routes of tumorigenicity (teratomas and true tumors) and underlying mechanisms behind them were discussed. Finally, possible solutions to circumvent them were briefly introduced as well.

As compared to using pure stem cell-based therapeutic approach to facilitate neural regeneration, engineered biomaterial scaffolds exhibit a better prospective strategy, where grafted scaffold could provide a matrix support for cell penetration, proliferation, differentiation, and guidance to the target area. In L. Luo et al.'s another paper, the authors use DPSCs as the seeded cells in nerve tissue engineering and evaluate the effects of heparin-poloxamer (HP) hydrogel as a scaffold containing bFGF and DPSCs on the neural regeneration and functional recovery after spinal cord injury (SCI). The authors claimed that DPSCs combined with bFGF in HP-hydrogel (DPSCs-bFGF-HP) could enhance motor and sensory functional recovery after SCI than those of the other experimental groups. Increased expression of Bcl-2 and reduced expression of Bax and Caspase-3 to inhibit neuron apoptosis in the early process of recovery were also found. DPSCs-bFGF-HP could promote the expression of MBP and GAP43 during nerve repair process after SCI. As a result, the combination of HP hydrogel, DPSCs, and bFGF could be applied as an effective treatment for neuron regeneration and tissue restoration after SCI in the future. In L. Zhou et al.'s paper, poly(lactic-co-glycolic acid) (PLGA) scaffold was utilized to support mesenchymal stem cell (MSC) and neuron growth *in vitro* and *in vivo*. In particular, the effect of PLGA scaffold on cell proliferation and differentiation of MSC towards neurogenesis were assessed. This study suggested that MSCs-PLGA complex may be used as tissue engineering material for brain injury.

In this interdisciplinary research field of neural tissue regeneration, these papers show us exciting outcomes and insightful perspectives with state-of-the-art in rising topics. We hope that this special issue would be of great concern to the interested readers. We also would like to express our great appreciation to all the authors, reviewers, and the editors for their support in making this special issue possible.

